# Immune Cells in Cutaneous Wound Healing: A Review of Functional Data from Animal Models

**DOI:** 10.3390/ijms23052444

**Published:** 2022-02-23

**Authors:** David M. Chesko, Traci A. Wilgus

**Affiliations:** Department of Pathology, Ohio State University, Columbus, OH 43210, USA; chesko.8@osu.edu

**Keywords:** immune cells, inflammation, skin, wound healing, repair

## Abstract

The healing of skin wounds involves the activation and recruitment of various immune cell types, many of which are believed to contribute significantly to different aspects of the repair process. Roles for immune cells have been described in practically all stages of wound healing, including hemostasis, inflammation, proliferation and scar formation/remodeling. Over the last decade, tools to deplete immune cell populations in animal models have become more advanced, leading to a surge in the number of studies examining the function of specific immune cell types in skin repair. In this review, we will summarize what is known about distinct immune cell types in cutaneous wound healing, with an emphasis on data from animal studies in which specific cell types have been targeted.

## 1. Introduction

Immune cells play an active role in cutaneous wound repair. These cells are important for combating infection, but they also influence repair by producing a variety of cytokines, growth factors, enzymes and other mediators that regulate both the activity of surrounding cells as well as the synthesis and remodeling of extracellular matrix (ECM) molecules. The repair process is often described as a series of successive phases (albeit with some amount of temporal overlap) that include hemostasis, inflammation, proliferation, and scar formation/remodeling. Each of these phases can be affected by the presence or activity of different immune cells [1,2,3,4]. This review will discuss published papers utilizing antibody-based and genetic methods to deplete specific subsets of immune cells to study their function in the cutaneous wound healing process. Included are studies that have examined the function of immune cells belonging to the myeloid lineage (platelets, mast cells, neutrophils, and macrophages) and the lymphoid lineage (T cells, B cells and innate lymphoid cells) in wound healing.

## 2. Myeloid Cells in Wound Healing

Cells of the myeloid lineage make up a large part of the innate immune system and most myeloid cells play an important role in preventing infection and stimulating the adaptive branch of the immune system [5]. The effects of depleting red blood cell-derived platelets, as well as mast cells, neutrophils, and macrophages on wound healing have been assessed in animal models. More recent studies have also started to look more closely at the function of other myeloid cells, such as dendritic cells, in the repair process. 

### 2.1. Platelets

Platelets (also known as thrombocytes) are small, non-nucleated blood cells produced from megakaryocytes. They accumulate rapidly in injured tissues, where they primarily function in hemostasis [2,6]. Exposure to subendothelial collagen in damaged blood vessels and other stimuli present in injured tissue causes platelet aggregation and activation. This stimulates the clotting process and the formation of fibrin, which helps stop blood loss from damaged vessels. Fibrin produced in the wound bed during early stages also acts as a provisional matrix that facilitates the migration of cells into the wound [7,8]. In addition to hemostatic functions, platelets also have various immune functions and serve as a rich source of growth factors that can stimulate the activity of other cell types involved in repair, including platelet-derived growth factor (PDGF), vascular endothelial growth factor (VEGF) and transforming growth factor-beta (TGF-β) [6,9,10].

Although the importance of platelets in initiating hemostasis and secreting growth factors is well established, studies in mice have suggested that the absence of platelets has little effect on the outcome of normal wound healing in excisional wounds [11]. Depletion of platelets via injection of anti-platelet serum did not have a strong effect on neutrophil recruitment, but it did cause an increase in MOMA-2^+^ macrophages 2–3 days after injury and CD-3^+^ T cells at 10 days. Although platelet depletion enhanced the recruitment of macrophages and T cells, it had no significant effects on the rate of wound re-epithelialization, collagen synthesis, granulation tissue area, or vascular density [11]. It is possible that in the absence of platelets, mediators produced by other cells are able to overcome the lack of platelet-derived mediators in thrombocytopenic mice.

Although there have not been many animal studies targeting platelets, it should be noted that platelets have been suggested as a possible therapeutic option for chronic wounds. For example, platelet-rich plasma (PRP) therapy has been discussed as a treatment option for difficult to heal wounds, providing a means to deliver growth factors and stimulate healing [12]. PRP has undergone some testing in human wounds. Although the studies are limited and the results have been somewhat variable, the approach appears to be promising [13,14,15,16]. Additional in-depth clinical studies will have to be performed to determine the usefulness of PRP for wound healing in humans. 

### 2.2. Mast Cells 

Mast cells are resident inflammatory cells in the skin that quickly become activated and degranulate after injury, then increase in numbers over time during the healing process. Upon degranulation, they release many pre-stored mediators and eventually synthesize and release additional mediators that can influence repair. Mast cells have been suggested to play a role in several phases of wound healing [17,18,19,20]. The most well studied functions of mast cells in wound healing are in neutrophil recruitment, wound closure, and scar formation, but the results have varied based on the methods used to examine mast cell function. 

To study the role of mast cells in wound healing, a number of mast cell-deficient mouse strains have been used (Table 1). Kit^W/W-v^ and Kit^W-sh/W-sh^ strains have mutations in the *kit* gene, which encodes a tyrosine kinase receptor required for proper mast cell development. Because mice with *kit* mutations can have other abnormalities and cells other than mast cells can be affected, newer mast cell-deficient strains have been developed. These include: Cpa3-Cre/Mcl-1^fl/fl^ mice, which lack mast cells due to cre recombinase-mediated ablation of the anti-apoptotic factor Mcl-1 (myeloid leukemia cell differentiation protein-1) in carboxypeptidase A (Cpa3)-expressing mast cells [21]; Cre-Master Cpa3^Cre/+^ mice, which are mast cell-deficient due to cre-related genotoxicity in Cpa3-expressing mast cells [22]; Mcpt5-Cre/iDTR mice, in which diphtheria toxin can be used to induce deletion of mast cell protease 5 (Mcpt5)-expressing mast cells [23]; and Mas-TRECK (Mast cell-specific enhancer-mediated Toxin Receptor-mediated Conditional cell Knock out) mice, in which diphtheria toxin can be used to ablate mast cells due to the expression of human diphtheria toxin receptor being driven by an intronic enhancer element of the Il-4 gene [24,25].

Mast cells produce a large number of mediators that are capable of stimulating other immune cells. Initial studies in Kit^W/W-v^ mice showed that the mast cell-deficient mice contained significantly fewer neutrophils compared to wild-type mice [26,27], suggesting that mast cells help recruit neutrophils to the wound site. However, two more recent studies using newer mast cell-deficient strains have reported no changes in neutrophil infiltration into wounds in the absence of mast cells [28,29]. 

The data on whether mast cells promote wound closure varies. Studies of normal wound healing in Kit^W/W-v^ mice have reported delayed wound closure in mast cell-deficient mice [27], but others have suggested wound closure is unaffected in the absence of mast cells [26,30,31]. Studies in other strains of mast cell-deficient mice have also reported no significant differences in wound closure in mice lacking mast cells [28,29,31]. However, studies using models of more complex wounds have yielded different results. In diabetic wounds mast cell deficiency due to *kit* mutations appears to delay wound closure [32] and in an infected wound model both Kit^W/W-v^ and Cpa-Cre;Mcl-1^fl/fl^ mast cell-deficient mice were shown to heal with increased microbial burden and delayed healing [33].

Results related to the importance of mast cells in collagen deposition and scar formation have also been inconsistent. Some results have supported a role for mast cells in promoting scar formation. In a thermal injury model, reduced dermal thickness and wound edge fibrosis were observed in Kit^W/W-v^ mice [34]. In scar-forming incisional fetal skin wounds, Kit^W/W-v^ mice healed with less scarring [35]. Other studies in Kit^W/W-v^ mice have indicated that a lack of mast cells leads to alterations in collagen maturation and less granulation tissue formation [36] and less interwoven collagen at the edges of the wound bed [30]. However, other studies have indicated that mast cells do not play a strong role in the dermal repair process. One study using splinted excisional wounds showed no difference in collagen density or scar size in mast cell-deficient Kit^W/W-v^, Kit^W-sh/W-sh^, or Cpa3-Cre/Mcl-1^fl/fl^ mice [31]. Studies in Cpa3^Cre/+^ and Mcpt5-Cre/iDTR mice also indicated that mast cell deficiency did not affect scar size [28,29]. 

In addition to mast cell-deficient mouse strains, chemicals that prevent degranulation (i.e., mast cell stabilizers) such as disodium cromoglycate (DSCG) and ketotifen have been used to study mast cells in wound healing. In normal wounds, mast cell stabilizers do not appear to significantly alter wound closure kinetics. In mice treated with DSCG [37] and in pigs treated with ketotifen [38], mast cell stabilizers did not significantly affect the rate of re-epithelialization. However, in diabetic wound models which are characterized by delayed healing, treatment with DSCG [32] or the novel mast cell stabilizer MCS-01 [39] accelerated wound closure. Some studies have also examined the effects of mast cell stabilizers on collagen deposition and scar formation. Injection of DSCG into rat wounds was reported to reduce collagen levels [40] and systemic treatment with DSCG was shown to reduce scar size and normalize the density of collagen fibrils and overall collagen architecture in mouse wounds [37]. Another study examined the effects of ketotifen in red Duroc pigs, which is used as a large animal model of hypertrophic scarring [38]. The authors reported reduced wound contraction and scar formation in pigs treated with ketotifen. These scars also contained thinner and less dense collagen fibers and fewer myofibroblasts [38]. While mast cell stabilizers can provide some useful information about the role of mast cells, these drugs may affect other cell types. 

Overall, the lack of consistent results with regard to the role of mast cells in wound healing and scar formation makes it difficult to precisely define their function. More specific depletion methods and more standardized assessments of healing/scar formation may be needed to gain better insights into mast cell-dependent aspects of cutaneous healing.

### 2.3. Neutrophils

Neutrophils are circulating inflammatory cells that are recruited to the wound site quickly after an injury. They are often considered the first line of defense against infection because their numbers peak at the wound site early after injury, in contrast to monocyte/macrophage numbers which generally peak several days later [2]. Systemic, antibody-based neutrophil depletion studies have suggested that neutrophils do not have striking positive effects on normal wound healing, but that they are important in infected wounds (Table 2). One of the first studies to examine the role of neutrophils in wound healing was by Simpson and Ross [43]. In this study, the authors used an antibody-based approach (anti-neutrophil serum) to deplete neutrophils in incisional wounds in guinea pigs. At early time points there were no differences in monocytes or fibrin deposition and at later stages of healing there were no differences in repair, cellularity, or amount of connective tissue deposition. This suggested that neutrophil depletion does not impair healing in normal wounds [43]. In a later study by Dovi and colleagues, antibody-based neutrophil depletion did not significantly affect the number of wound macrophages or collagen deposition [44]. Interestingly, neutrophil depletion enhanced the rate of re-epithelialization in both normal and diabetic mice, suggesting that under some circumstances neutrophils may delay healing [44]. This is in line with some human studies showing an increase in the number and/or an extended presence of neutrophils in poorly healing chronic wounds (reviewed in [45]). Neutrophils do, however, appear to be important in infected wounds. A more recent study has shown that neutrophil depletion leads to more severe infection and delayed wound closure in *A. baumannii*-infected wounds [46]. It seems that an appropriate number of neutrophils is needed for optimal healing and that the ideal number is dependent on the wound microenvironment. In an infected wound neutrophils are likely needed to help eliminate infection and allow healing to proceed, whereas an overactive neutrophil response may have negative consequences due to the release of destructive proteases and reactive oxygen and nitrogen species [47].

### 2.4. Macrophages

Macrophages are resident inflammatory cells that play an important role in wound healing [48] (Table 3). Macrophage numbers increase several days after injury, in part due to the recruitment of circulating monocytes and their subsequent differentiation into macrophages. Initial studies investigating the role of macrophages in wound healing performed by Leibovich and Ross suggested these cells were important for wound healing [49]. They used a combination of hydrocortisone and anti-macrophage serum to deplete monocytes/macrophages in guinea pigs and reported delayed healing and reduced collagen deposition. However, the use of hydrocortisone to more fully deplete the monocytic cell population complicates the interpretation of the results. Several decades later, genetic depletion models started to become more widely used. One study by Martin and colleagues performed in mice lacking the ETS-family transcription factor PU.1, which is involved in hematopoietic cell differentiation, suggested that a lack of macrophages does not cause a delay in healing, but may lead to healing with less scar formation [50]. However, these mice have reduced numbers of some other immune cells, making it difficult to pinpoint the specific role of macrophages.

More advanced depletion methods have suggested that macrophages are critical for proper wound repair. Three separate groups using diphtheria toxin-based genetic models have shown that macrophage depletion leads to significant delays in wound closure [51,52,53], with concomitant reductions in collagen deposition, myofibroblasts, and angiogenesis (depending on the model used). Additional studies by Shook and colleagues validated these results and suggested that a specific subset of macrophages expressing CD301b may be responsible for the delayed healing observed in macrophage-depleted mice [54]. The results from genetic depletion models have consistently pointed to macrophages as a key pro-healing cell type.

In addition to antibody- and genetically-based depletion methods, treatment with clodronate liposomes has also been used as a way to deplete tissue macrophages. Using this method, Zhu et al. showed that clodronate-mediated macrophage depletion reduced scar formation in a hypertrophic scar model [55]. While the studies using genetic depletion methods discussed above did not examine scar formation in detail, several did report reduced collagen deposition and/or myofibroblast numbers, suggesting macrophages may stimulate scar formation. More thorough studies that assess the amount of scar tissue and the structure/organization of the collagen deposited are needed to define the role of macrophages and specific macrophage subtypes in scar formation.

### 2.5. Other Myeloid Cells

In addition to the cell types discussed above, there are other myeloid-derived cells that could play a role in wound healing, such as basophils, eosinophils, and dendritic cells. There is less known about the specific role these cells play in wound healing, in part because cell-specific depletion can be challenging. However, several tools have been developed in recent years to study the role of dendritic cells (DCs) in vivo. DCs are important antigen presenting cells that can be divided into several subtypes, including plasmacytoid DCs, monocyte-derived DCs, conventional DCs (type 1 and 2) and Langerhans cells. Only a few functional DC studies have been performed. One study suggested that diphtheria toxin-mediated depletion of langerin-expressing cells (Langerhans cells and dermal langerin^+^ DCs [56]) accelerates wound closure [57]. Another study suggested that depletion of CD11c-expressing cells (conventional and plasmacytoid DCs [56]) delays wound closure [58]. Additional studies in burn wounds have suggested that increasing the number of DCs enhances the ability of the immune system to eliminate infection, and this appears to be at least partially mediated by plasmacytoid DCs [59,60,61]. It seems that different subtypes of DCs may have distinct functions in wound healing based on the limited number of functional studies that have been performed, but additional work is needed.

## 3. Lymphoid Cells in Wound Healing

Cells of the lymphoid lineage include typical cells of the adaptive immune system (T cells and B cells), as well as innate lymphoid cells (ILCs), which are derived from the lymphoid lineage but share features with innate immune cells. Thus far, most published studies on lymphoid cells in wound healing have focused on T cells; however, several recent studies have suggested B cells and ILCs may regulate various aspects of repair.

### 3.1. T Cells

T cells develop in the thymus and differentiate into various effector cell types that help recognize and eliminate pathogens and regulate immune responses. A number of studies have suggested that T cells play a role in several phases of wound healing healing (Table 4). T cells can generally be divided into two main subtypes: αβ T cells and γδ T cells, depending on the type of T cell receptor (TCR) they express. The roles of several other types of specialized T cells, such as invariant NKT cells (iNKT) and mucosal-associated invariant T cells (MAIT), in wound healing have also been studied.

Several groups have studied the effects of pan T cell depletion on wound healing. Early work by Peterson et al. [62] used anti-Thy1.2 antibodies in conjunction with an incisional wound and PVA sponge implant to study the role of T cells in repair. The authors reported reduced breaking strength in the healed wound and less collagen deposition in the PVA sponges, suggesting T cells stimulate collagen production [62]. However, the use of anti-Thy1.2 antibodies as a tool for T cell depletion complicates the interpretation of the results, as we now know that there are many T cell subtypes with opposing functions and anti-Thy1.2 antibodies may deplete other types of T cells (e.g., Thy1.2^+^ γδ T cells) and some cells of the lymphoid lineage (e.g., ILCs). Later studies using the PVA model attempted to tease out the effects of different T cell subsets by comparing depletion with anti-Thy1.2 to that of depletion with a combination of anti-CD4 and anti-CD8 antibodies [63]. Similar to prior work, anti-Thy1.2 antibodies led to reduced wound breaking strength and less collagen in the PVA sponges. However, the combination of anti-CD4 and anti-CD8 antibodies increased wound breaking strength and collagen deposition [63], suggesting that a CD4-/CD8- population of cells expressing Thy1.2 is important for wound healing. It has been suggested that this cell population may be γδ T cells [64].

Other studies have used athymic nude mice to determine whether the absence of T cells affect wound healing, and the results have been somewhat variable. Barbul and colleagues reported an increase in wound breaking strength and an increase in collagen deposition in PVA sponges in athymic nude mice [65], whereas studies by Gawronska-Kozak et al. suggested that nude mice heal with reduced collagen deposition and reduced scar formation in incisional wounds compared to normal mice [66]. In the study by Gawronska-Kozak and colleagues, multiple T cell-deficient mouse models were compared. Nude mice healed with the least amount of scar tissue compared to the other groups and they also had low levels of inflammation and high levels of hyaluronan. One distinguishing feature in the nude mice compared to the others was the relative lack of CD8+ T cells in the nude mice which healed with minimal scarring [66]. Alternative approaches, such as adoptive transfer of various T cell subsets in immunodeficient strains, are also being performed to test the function of various T cell populations on scar formation [67] but more work needs to be done to improve our understanding of T cells in this process.

#### 3.1.1. αβ T Cells

αβ T cells are the most widely studied T cell subpopulation in wound healing. αβ T cells are often subdivided based on the presence of CD4 or CD8 molecules. CD4^+^ T cells, or helper T cells, primarily serve to regulate immune responses, whereas the main function of CD8^+^ T cells (cytotoxic T cells) is to kill abnormal cells (e.g., tumor cells or infected cells). Several studies have depleted CD4^+^ T cells, CD8^+^ T cells, or both CD4^+^ and CD8^+^ T cells. In studies using antibody-mediated depletion, the results have been variable. One group reported that anti-CD4 antibodies had no effect on collagen deposition or wound breaking strength [68], whereas another reported reduced wound breaking strength [69]; however, both groups showed an increase in wound breaking strength with anti-CD8 antibody treatment. In studies where both anti-CD4 and anti-CD8 antibodies were administered, one group reported an increase in collagen deposition and wound breaking strength [63] while another reported no change in wound breaking strength [69]. In a more thorough study comparing mice that are genetically deficient in either CD4 or CD8, no changes in wound closure/reepithelialization, wound breaking strength, collagen deposition, or angiogenesis were observed in either knockout strain; however, CD4 knockout mice had an increase in CD8^+^ cells and CD8 knockout mice had a decrease in CD4^+^ cells as well as neutrophils and macrophages [64]. The lack of consistency between studies on CD4^+^ and CD8^+^ depletion suggests that temporal and more precise depletion of specific T cell subsets may generate more useful information.

One subset of CD4^+^ T cells, regulatory T cells (Treg), is known to play a role in dampening immune responses. Two recent studies have examined the role of these cells in wound healing by depleting them with diphtheria toxin in foxp3-DTR transgenic mice [70,71]. Both groups observed delayed wound closure and an increase in αβ T cells in Treg-depleted mice [70,71], suggesting that proper modulation of the immune response after injury is needed for optimal healing.

Other specialized αβ T cell subsets with innate immune cell properties, such as iNKT and MAIT cells have also been studied in wound healing. iNKT cells express an invariant form of the α chain of the TCR and typically recognize lipid antigens bound to CD1. Studies on iNKT cells in wound healing have generated conflicting results in excisional wound models. One group suggested that mice lacking either iNKT cells or the ability of iNKT cells to be activated (Jα281 knockout mice or anti-CD1 antibody-treated mice, respectively) heal more quickly [72,73], whereas another group suggested mice lacking iNKT cells (Jα281 knockout mice) heal more slowly in both infected and uninfected wounds [74,75,76]. It is unclear what accounts for these discrepancies, although it has been suggested that differences in background mouse strains or treatment of the wounds (covered with dressings or not) may have played a role [75]. MAIT cells express semi-invariant forms of the αβ TCR and murine studies have shown that variations in microbial exposure early in life affect the number of MAIT cells populating the skin [77]. Mice exposed to *S. epidermidis* that lacked the MHC-Ib molecule MR1, which have fewer MAIT cells, were shown to have slower epithelial closure of excisional wounds as measured by epithelial tongue length. In addition, application of the riboflavin derivative 5-(2-oxopropylideneamino)-6-D-ribitylaminouracil (5-OP-RU), which increases MAIT cells, led to an increase in epithelial closure [77]. Additional studies are needed to fully understand the role of unconventional αβ T cell subsets in wound healing.

#### 3.1.2. γδ T Cells

γδ T cells express a unique TCR with γ and δ chains rather than the typical α and β chains present on conventional T cells. γδ T cells are involved in immune surveillance and regulation of tissue homeostasis [78], and different γδ T cell populations are present in the epidermal and dermal layers of the skin. γδ T cells in the epidermis of mice, which are known as dendritic epidermal T cells (DETCs), express an invariant Vγ5Vδ1 TCR (Vγ5^+^ cells) [79]. On the other hand, γδ T cells in the dermis typically express either the Vγ6Vδ1 (Vγ6^+^ cells) or Vγ4Vδ4 (Vγ4^+^ cells) TCR [79] (TCR chain descriptions are consistent with Heilig nomenclature).

DETCs are known to respond to factors released by damaged or stressed keratinocytes, and change their morphology upon activation in response to skin injury [79]. Studies by Jameson et al. were the first to demonstrate the functional importance of γδ T cells in wound healing [80]. They demonstrated delayed wound closure and reduced keratinocyte proliferation in excisional wounds from TCRδ^−/−^ knockout mice, which lack DETCs [80]. TCRδ^−/−^ knockout mice also display an increase in mortality and altered growth factor and cytokine production after burn wound healing [81,82]. Additional studies have shown that γδ T cells in the epidermis promote healing by producing FGF-7, FGF-10, IGF-1, IL-17a [80,83,84,85]. They also stimulate the production by hyaluronan by keratinocytes, which then aids in the recruitment of macrophages to the wound [86].

Less is known about the function of dermal γδ T cells in wound healing. Results from one study suggested that the activity of Vγ4^+^ dermal γδ T cells has a negative effect on wound closure, in part by counteracting the pro-healing actions of DETCs [87]. Another study by Gay et al. focused on understanding the role of γδ T cells in wound-induced hair follicle neogenesis (WIHN) [88]. They showed that TCRδ^−/−^ knockout mice had defective WIHN, and that dermal γδ T cells stimulate WIHN by producing FGF-9, which subsequently stimulates Wnt activation [88].

### 3.2. B Cells

B cells develop in the bone marrow and are an important part of the adaptive immune response. They are critical for the production of antibodies and their ability to communicate with other immune cells is important for preventing infection. B cells are often overlooked in studies focused on the skin, but recent reports have highlighted the importance and the complexity of these cells. It is now recognized that B cells are present in healthy skin where they help maintain homeostasis, and, like many other immune cell types, there are different B cell subsets capable of either promoting or suppressing cutaneous inflammatory responses depending on the circumstances [89]. With regard to wound healing, studies have reported an increase in B cells in both human and rodent skin wounds [90,91]. To our knowledge, there are no published wound healing studies that have specifically depleted B cells; however, several studies have suggested a role for B cells in the healing process. Iwata and colleagues examined wound healing in mice that either lacked or overexpressed CD19, an important B cell regulatory molecule [92]. Excisional wounds in CD19-deficient mice healed more slowly compared to wild-type controls, whereas CD19 overexpressing mice healed more quickly. CD19-deficient mice also had fewer B cells, so the delay in healing could have resulted from reduced B cell numbers and/or activation. Additional experiments in this paper suggested that hyaluronic acid accelerates wound healing by stimulating TLR4, and that this effect is dependent on CD19 [92]. Another study by Sirbulescu et al. showed that the application of mature B cells to splinted excisional wounds in either normal or diabetic mice lead to faster wound closure [93]. Interestingly, a beneficial effect was not observed when the same number of T cells were applied to wounds. Although studies specifically exploring B cell function in skin wounds are limited, the data suggest that B cells may be beneficial for wound repair.

### 3.3. Innate Lymphoid Cells (ILCs)

ILCs are members of the lymphoid lineage; however, unlike T and B cells, they do not have antigen-specific receptors and are therefore considered innate, rather than adaptive, immune cells. ILCs include lymphoid tissue-inducer cells and natural killer (NK) cells, as well as ILC1, ILC2, and ILC3 cells [94]. There is very little information about ILCs in cutaneous wound healing, but a few studies have suggested that some ILC subsets play a role.

NK cells are traditionally known for their ability to detect and kill tumor and virus-infected cells, but studies by Sobecki et al. suggested that NK cells are also active during wound healing [95]. Depletion of NK cells in excisional wounds using anti-NK1.1 antibodies led to an early acceleration in wound closure via macroscopic measurement along with a corresponding increase in blood vessel density. Conditional ablation of HIF-1a in cells expressing the gene encoding NKp46 (Ncr1, primarily expressed by NK cells) similarly lead to accelerated wound closure and an increase in vascularity, which may have been due to reduced production of IFN-γ and GM-CSF by HIF-1a-deficient NK cells. Interestingly, when challenged with group A *Streptococcus*, these mice had a dampened antimicrobial response [95]. Taken together, the results suggest that NK cells may not be beneficial for wound healing under normal circumstances, but that they likely aid in the response to infection.

There is also some evidence that ILC2 and ILC3 cells may play a role in skin repair. ILC2 cells are known to express the transmembrane form of the IL-33 receptor ST2, ST2L, and respond to IL-33 [96] which is upregulated after injury [97,98]. A study by Rak et al. showed that ILC2 cells increase in murine skin wounds, and ILC2-like cells (ST2L-expressing cells with a lymphoid morphology) were also found to be upregulated in human wounds [97]. Wounds in mice lacking IL-33 had reduced numbers of ILC2 cells and delayed wound closure, whereas intraperitoneal administration of recombinant IL-33 increased the number of ILC2 cells and accelerated wound closure. The positive correlation between ILC2 numbers and wound closure rates suggested that ILC2 cells may promote healing. Another group, Li and colleagues, examined ILC3 cells in wound healing [99]. They found that RORγ^+^ ILC3s were recruited to wounds in murine and human skin through a mechanism involving upregulation of TNF-α by activated epithelial Notch1. RORγ^−/−^ mice, which have reduced numbers of ILC3s, exhibited delayed gross wound closure, which was associated with a decrease in macrophages. Therefore, the presence of ILC3s may be beneficial for healing. Taken together, the studies suggest that ILCs play a role in the repair of skin wounds. Providing further support for this idea are experiments showing that ILC-depleted mice exhibit delayed healing. Administration of anti-CD90.2 (Thy1.2) antibodies in RAG^−/−^ mice was used to deplete ILCs in a splinted excisional wound model [97]. ILC-depleted mice showed delayed reepithelialization and wound closure compared to non-depleted mice (RAG^−/−^ mice treated with IgG control antibodies), indicating that ILCs may support wound healing. Further work and more specific methods of depleting individual types of ILCs are needed to gain a better appreciation for how each ILC subset contributes to the different phases of wound healing.

## 4. Conclusions

It is clear from reviewing the wound healing literature that an enormous amount of effort has been put into teasing out the function of various immune cells in the cutaneous repair process. The information in this area is growing at a rapid pace as new methods are developed to identify and target different immune cell populations in vivo. Unfortunately, the results do not always yield a clear understanding about the role individual immune cell types play in wound repair.

There are several possible explanations for the lack of consistency in the literature regarding the importance of immune cells in wound healing. There are a wide range of injury models (thermal, excisional, excisional with splinting, incisional, diabetic or non-diabetic, infected or non-infected), methods to measure healing (external wound closure assessments and various microscopic methods), cell depletion methods, and time points used in the studies described here, which could all affect the results and make it more difficult to make comparisons between studies. In addition, many of the depletion strategies that are currently used are not absolutely specific for one immune cell type. Because multiple subsets of each type of immune cell exist, the phenotype of many immune cells can change over time depending on microenvironmental cues, and other cell types may have the capacity to compensate when one population is missing, dissecting the specific contributions of individual cell types becomes an even more difficult task. Additional investigations will have to be performed in the future as better strategies to target specific subtypes are developed. In the process of advancing current techniques to study immune cells in animal models, thought should also be given to how well the model replicates what might happen in human wounds, as both differences in the immune cell types and function as well as some inherent differences in the wound healing process itself exist between animals and humans. Overall, based on the current literature, it is clear that the immune response to wound healing is a complex process and that more work is needed to fully understand how individual immune cell types (and subtypes) contribute to the cutaneous repair process.

## Figures and Tables

**Table 1:** Summary of data from mast cell depletion papers.

Study	Wound Type	Targeting Method	Key Results
Egozi (2003) [26]	Excisional	Genetic (Kit^W/W-v^)	*No change:* - Reepithelialization (microscopic) - Vascularity (CD-31) - Collagen (hydroxyproline) - Macrophages (MOMA-2) - T cells (CD-3) *Decrease:* - Neutrophils (GR-1)
Iba (2004) [30]	Excisional	Genetic (Kit^W/W-v^)	*No change:* - Wound closure (macroscopic) *Increase:* - Collagen (hydroxyproline)
Weller (2006) [27]	Excisional	Genetic (Kit^W/W-v^)	*Decrease:* - Wound closure (macroscopic) - Neutrophils (MPO activity) - Vascular permeability (Evans blue)
Shiota (2010) [34]	Thermal (2nd degree scald)	Genetic (Kit^W/W-v^)	*No change:* - Wound closure (macroscopic) - Reepithelialization (microscopic) *Decrease:* - Dermal thickness (microscopic) - Wound edge fibrosis (trichrome)
Younan (2010) [41]	Thermal (2nd degree scald)	Genetic (Kit^W/W-v^)	*Decrease:* - Erythema (macroscopic) - Ulceration (macroscopic) *Increase:* - Hair re-growth (macroscopic)
Younan (2011) [36]	Excisional +/micro-deformation	Genetic (Kit^W/W-v^)	*No change:* - Collagen (trichrome) *Decrease:* - Granulation tissue - Cell proliferation (Ki-67) - Vascularity (CD-31) - Collagen maturation (Herovici)
Wulff (2012) [35]	Incisional (fetal skin)	Genetic (Kit^W/W-v^)	*Decrease:* - Scar size (microscopic)
Antsiferova (2013) [28]	Excisional	Genetic (Cre-Master - Cpa3^Cre/+^)	*No change:* - Wound area (macroscopic) - Reepithelialization (microscopic) - Granulation tissue - Neutrophils (MPO activity)
Nauta (2013) [31]	Splinted excisional	Genetic (Kit^W-sh/W-sh^; Kit^W/W-v^; Cpa3-Cre;Mcl-1^fl/fl^)	*No change:* - Wound size (macroscopic) - Scar area (microscopic) - Collagen density (trichrome)
Willenborg (2014) [29]	Excisional	Genetic (Mcpt5-Cre/iDTR)	*No change:* - Wound size (macroscopic) - Reepithelialization (microscopic) - Granulation tissue - Myofibroblasts (α-SMA) - Collagen organization (picrosirius red) - Neutrophils (Ly-6G/CD-11b) - Macrophages (F4/80/CD-11b) *Decrease:* - Early panniculus carnosus - contraction (microscopic)
Tellechea (2016) [32]	Diabetic excisional	Genetic (Kit^W/W-v^; Kit^W-sh/W-sh^)	*Increase:* - Wound size (macroscopic) Decrease: - Reepithelialization (microscopic) ^†^
Nishida (2019) [42]	Excisional	Genetic (Kit^W-sh/W-sh^; Mas-TRECK)	*Increase:* - Wound size (macroscopic)
Zimmermann (2019) [33]	Infected excisional	Genetic (Kit^W/W-v^; Cpa-Cre;Mcl-1^fl/fl^)	*Increase:* - Bacterial burden - Wound size (macroscopic) *

^†^ shown for Kit^W/W-v^ mice only. * no change in uninfected wounds.

**Table 2:** Summary of data from neutrophil depletion papers.

Study	Wound Type	Targeting Method	Key Results
Simpson (1972) [43]	Incisional	Antibody (anti-neutrophil serum)	*No change:* - Monocytes - Cellularity - Repair - Connective tissue
Dovi (2003) [44]	Excisional	Antibody (anti-GR-1)	*No change:* - Collagen (hydroxyproline) - Macrophages (MOMA-2) *Increase:* - Reepithelialization (microscopic) ^†^
Grguric-Smith [46]	Infected excisional	Antibody (anti-Ly-6G)	*Decrease:* - Wound closure (macroscopic) *Increase:* - Bacterial burden - Collagen (RT-PCR, IHC)

^†^ Re-epithelialization was accelerated in both normal and diabetic neutropenic mice.

**Table 3:** Summary of data from macrophage depletion papers.

Study	Wound Type	Targeting Method	Key Results
Mirza (2009) [53]	Excisional	Genetic (CD11b-DTR)	*No change:* - Neutrophils (Ly-6G) - Monocytes (Ly-6C) - Collagen (hydroxyproline) - Wound disruption strength *Decrease:* - Reepithelialization (microscopic) - Granulation tissue - Collagen (trichrome) - Vascularity (CD-31) - Cellular proliferation (BrdU)
Goren (2009) [51]	Excisional	Genetic (LysM-cre/DTR)	*Increase:* - Wound size (microscopic) - Neutrophils (Gr-1) *Decrease:* - Contraction (microscopic) - Vascularity (CD-31) - Myofibroblasts (αSMA)
Lucas (2010) [52]	Excisional	Genetic (LysM-cre/iDTR)	*Increase:* - Wound size (macroscopic) *Decrease:* - Epithelial closure (microscopic) - Panniculus carnosus contraction (microscopic) - Granulation tissue - Scar formation (picrosirius red) - Vascularity (CD-31) - Myofibroblasts (α-SMA) *No change:* - Neutrophils (Gr-1)
Shook (2016) [54]	Excisional	Genetic (LysM-cre/iDTR; Mgl2^DTR/GFP^^†^)	*Decrease:* - Reepithelialization (microscopic) - Proliferation (phospho-histone H3) - Fibroblasts (ER-TR7) - Vascularity (CD-31) - Myofibroblasts (α-SMA) #

^†^ Mgl2^DTR/GFP^ mice lack CD301b+ cells. # shown for Mgl2^DTR/GFP^ mice only.

**Table 4:** Summary of data from T cell depletion papers.

Study	Wound Type	Targeting Method	Key Results
***Pan T cell depletion***			
Peterson (1987) [62]	Incisional + PVA sponge	Antibody (anti-Thy1.2)	*Decrease:* - Wound breaking strength - Collagen (hydroxyproline) *
Barbul (1989) [65]	Incisional + PVA sponge	Genetic (Foxn1^nu^)	*Increase:* - Wound breaking strength - Collagen (hydroxyproline) *
Efron (1990) [63]	Incisional + PVA sponge	Antibody (anti-Thy1.2)	*Decrease:* - Wound breaking strength - Collagen (hydroxyproline) *
Gawronska-Kozak (2006) [66]	Incisional	Genetic (Foxn1^nu^)	*Decrease:* - Scar formation (trichrome) - Collagen (hydroxyproline)
***αβ T cell depletion***			
Barbul (1989) [68]	Incisional + PVA sponge	Antibody (anti-CD4)	*No change:* - Wound breaking strength - Collagen (hydroxyproline) *
		Antibody (anti-CD8)	*Increase:* - Wound breaking strength - Collagen (hydroxyproline) *
Efron (1990) [63]	Incisional + PVA sponge	Antibody (anti-CD4 + anti-CD8)	*Increase:* - Wound breaking strength - Collagen (hydroxyproline) *
Davis (2001) [69]	Incisional + thymectomy	Antibody (anti-CD4)	*Decrease:* - Wound breaking strength
		Antibody (anti-CD8)	*Increase:* - Wound breaking strength
		Antibody (anti-CD4 + anti-CD8)	*No change:* - Wound breaking strength
Chen (2014) [64]	Excisional	Genetic (CD4^−/−^)	*No change:* - Wound closure (macroscopic) - Keratinocyte proliferation (Ki-67) - Wound breaking strength - Collagen (hydroxyproline) - Vascularity (CD-31) *Increase:* - CD8 T cells
		Genetic (CD8^−/−^)	*No change:* - Wound closure (macroscopic) - Keratinocyte proliferation (Ki-67) - Wound breaking strength - Collagen (hydroxyproline) - Vascularity (CD-31) *Decrease:* - CD4 T cells - Neutrophils (Gr-1) - Macrophages (CD68)
***Treg depletion***			
Nosbaum (2016) [71]	Excisional	Genetic (Foxp3-DTR)	*Decrease:* - Wound closure (macroscopic) *Increase:* - Proinflammatory macrophages (Ly-6Chi) - CD4 and CD8 T cells
Haertel (2018) [70]	Excisional	Genetic (Foxp3-DTR-eGFP)	*Decrease:* - Wound closure (microscopic) - Reepithelialization (microscopic) - Contraction (microscopic) *Increase:* - αβ T cells
***γδ T cell depletion***			
Jameson (2002) [80]	Excisional	Genetic (TCRδ^−/−^)	*Decrease:* - Wound closure (macroscopic) - Keratinocyte proliferation (BrdU)

* Measured in PVA sponge.

## Data Availability

Not applicable.

## Abbreviations

α-SMA	Alpha-smooth muscle actin
BrdU	Bromodeoxyuridine
CD	Cluster of differentiation
Cpa	Carboxypeptidase A
DC	Dendritic cell
DETC	Dendritic epidermal T cell
DSCG	Disodium cromoglycate
DT	Diphtheria toxin
DTR	Diphtheria toxin receptor
ECM	Extracellular matrix
FGF	Fibroblast growth factor
Foxp3	Forkhead box protein p3
GM-CSF	Granulocyte-monocyte colony stimulating factor
HIF	Hypoxia-inducible factor
IFN	Interferon
Ig	Immunoglobulin
IGF	Insulin growth factor
IHC	Immunohistochemistry
ILC	Innate lymphoid cell
iNKT	Invariant natural killer T cell
IL	Interleukin
MAIT	Mucosal-associated invariant T cells
Mas-TRECK	Mast cell-specific enhancer-mediated toxin receptor-mediated conditional cell knock out
Mcl	Myeloid leukemia cell differentiation protein
Mcpt	Mast cell protease
Mgl2	macrophage galactose N-acetylgalactosamine specific lectin 2
MOMA-2	Macrophages/monocytes antibody-2
MPO	Myeloperoxidase
NK	Natural killer
PDGF	Platelet-derived growth factor
PRP	Platelet rich plasma
PVA	Polyvinyl alcohol
RAG	Recombination activating
ROR	Retinoic acid receptor-related orphan receptor
RT-PCR	Reverse transcriptase-polymerase chain reaction
TCR	T cell receptor
TGF-β	Transforming growth factor-beta
TLR	Toll-like receptor
TNF	Tumor necrosis factor
Treg	Regulatory T cell
VEGF	Vascular endothelial growth factor
WIHN	Wound-induced hair follicle neogenesis
Wnt	Wingless-related integration site
5-OP-RU	5-(2-Oxopropylideneamino)-6-D-ribitylaminouracil
